# Effect of Heartfulness meditation program on perceived stress and satisfaction with life of female students

**DOI:** 10.3389/fpsyt.2023.1214603

**Published:** 2023-11-02

**Authors:** Pradeep K Gupta, Neetu Malhotra, Preeti Goel, Jayaram Thimmapuram, Prasanna Krishna

**Affiliations:** ^1^Institute of Chartered Accountants of India, New Delhi, India; ^2^Satyam Fashion Institute, Noida, India; ^3^Satyam College of Education, Noida, India; ^4^Internal Medicine, WellSpan York Hospital, York, PA, United States; ^5^Department of Research, Heartfulness Institute, Hyderabad, India

**Keywords:** female students, Heartfulness, meditation, satisfaction with life, stress

## Abstract

Though literacy levels are slowly improving in India due to the steps taken by the Government of India, it has been a challenge for female students with gender disparities in literacy levels. Stress levels are high in students, more so during their exam times. While prior literature has demonstrated the prevalence of stress during student life, there is limited evidence of any interventions to decrease stress and improve satisfaction with life, specifically for female students. Heartfulness meditation is a heart-based meditation practice that may reduce stress and improve satisfaction with life among female students. We conducted a prospective cohort analysis comparing perceived stress and satisfaction with life outcomes among female students who self-selected to participate in the Heartfulness meditation program. The first group, G20, underwent intervention from week 0 until week 12 and continued their practice independently until week 20. The second group, G14, joined the intervention from week 7 until week 12 with only a 6-week exposure to guided meditation sessions through Heartfulness Trainers. They continued their practice independently until week 20. Data on Perceived Stress (PSS) and Satisfaction with Life (SWLS) were collected at week 0 and week 20 for both groups. G20 had 127 participants, and G14 had 113 participants. Ninety-four participants from the G20 group and 93 from G14 completed the study. The G20 group showed a statistically significant reduction of PSS scores from 20.79 to 18.96, and an increase in the SWLS scores from 22.03 to 23.51 from baseline to the end of the study period. The study of the Pearson correlation coefficient indicated that there is a weak positive correlation between pre- and post-scores for both the groups for both PSS and SWLS. Our findings suggest that Heartfulness meditation practice offers significant benefits with perceived stress and satisfaction with life among female students independent of their age. Still, a prolonged guided intervention of over 6 weeks is needed. A larger, randomized study with an additional control group with no meditation intervention is recommended.

**Clinical trial registration:**

ISRCTN11302156, https://doi.org/10.1186/ISRCTN11302156.

## Introduction

Gender parity in elementary education has only reached 49% of the world’s nations. The gender disparity grows at the secondary level, where just 24% of countries have achieved gender parity in upper secondary education and 42% in lower school (1). Women’s literacy rate in India is 65% compared to the literacy rate of males at 82%. At its current state of progress, India will only attain universal literacy in 2060 (2, 3).

To address issues related to illiteracy and the related gender disparity, the Government of India has initiated the formation of universities specifically focused on female students, and this study is at one such university, which, amongst other disciplines, offers courses on Fashion Design, Mass Media, and Teacher Education.

Stress is a widespread problem across the cross-section of society. Some scientific research is done in this area – specifically for stress related to student life. The stress seems to be more during the exams and when the results are expected. Some of the studies have also looked at interventions to reduce stress among students successfully (4–13). The current study focuses on two parameters, PSS and SWLS, related to female students, which can help understand the intervention’s effects on the two factors. There is also rich evidence of stress being strongly correlated with satisfaction with life. Higher levels of stress lead to lower satisfaction with life resulting in strain on personal well-being with poor sleep quality, emotional and physical exhaustion, and family conflicts (14, 15).

A growing body of literature on meditative practices, including mindfulness, yoga, and other forms, have shown to effectively decrease exhaustion, fatigue, burnout, anxiety, depression, and physical illnesses and improve sleep (16–25). Additionally, meditation has also shown to be beneficial in improving emotional intelligence, leadership skills, ability to focus, developing higher levels of self-actualization, and performing under stress making these forms of self-care interventions as a possible means for enhancing overall wellness (26–34).

Despite growing concern about increasing stress among female students, there has been limited research exploring feasible self-care interventions to manage stress outside of traditional clinical settings. Notably, most literature examining self-care interventions for stress is limited to healthcare settings for physicians, nurses, and medical students alike (19, 22).

Heartfulness meditation is a simple heart-based meditation practice aimed at achieving a balanced state of mind resulting in higher efficiency and peace. Prior research on Heartfulness meditation for stress, burnout, etc., has demonstrated favorable outcomes within hospital settings. Studies involving accounting professionals across India and other countries also demonstrated that Heartfulness Meditation has a positive impact on SWLS and Burnout of professionals (35). A study on the effect of Heartfulness Meditation on high school students also demonstrated the benefits on managing loneliness through meditation by means of a randomized study (36).

It is, therefore, valuable to explore the benefits of meditative practices for stress and satisfaction with life among female students. While the objective of this study was to look at the overall benefits of a contemplative practice on the psychological well-being of female students, SWLS and PSS were chosen as specific parameters to measure the same. We hypothesized that engaging with a 20- and 14-week Heartfulness meditation program would decrease stress and improve satisfaction with life among female students at different degrees of impact.

## The practice of Heartfulness meditation

From the ancient traditions of the Orient, many different yogic disciplines like Jnana Yoga, Bhakti Yoga, Karma Yoga, and Raja Yoga have emerged, with each discipline having its method of enhancing overall well-being (37–39). One such technique is the Heartfulness Meditation based on Raja Yoga. One of the unique features of Heartfulness Meditation is an aspect called ‘Yogic Transmission’ which helps in the easy centering of practitioners and is predominant when meditating with a Certified Heartfulness Trainer. The practice is typically offered through certified Heartfulness meditation trainers. It comprises a morning session focusing on relaxation and meditation, an evening rejuvenation session that involves the removal of emotional impressions of the day, and a session at night for a deeper connection with oneself involving a short meditation session before sleep (37–39). This form of meditation has been evaluated in various healthcare settings, schools, and corporate wellness globally. It has been shown to improve physical health, psychological health, burnout, sleep quality, stress, and loneliness.

## Materials and methods

### Participants

The study was a 20-week prospective cohort analysis comparing perceived stress and satisfaction with life outcomes among female students who were self-selected to participate in the Heartfulness meditation program. The study was approved by the institutional review board of the Satyam Group of Institutions and affiliated with SNDT Women’s University, Mumbai (ID 0508/Research/SGI-HFN).

**Figure 1:**
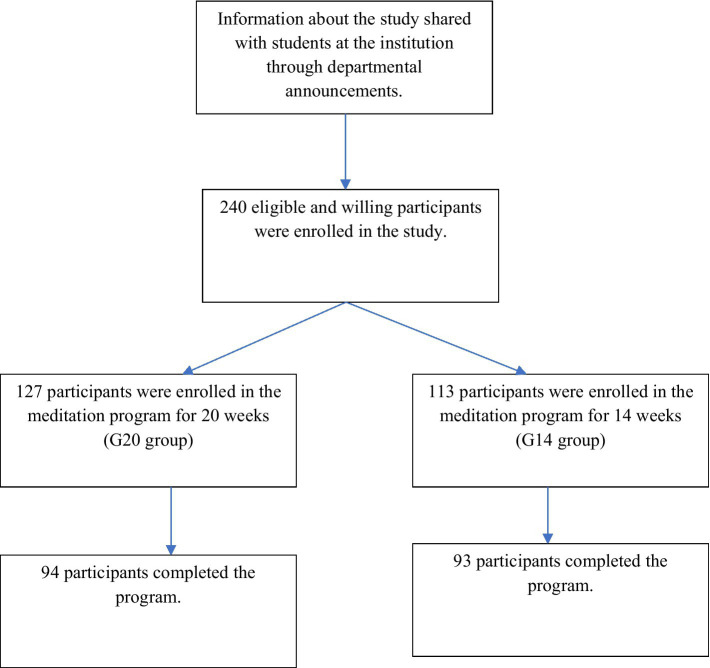
CONSORT Flow Chart.

Participants were recruited through communication from the institution to all students. Recruiting material included a direct online link for participants to review the study concept in detail, offer voluntary consent to participation, and self-register. While any student aged 18 years and above was eligible to participate, individuals with active suicidal ideation, current or past diagnoses of manic-depressive disorders, post-traumatic stress disorder, psychotic disorders, or any other psychiatric conditions requiring treatment were informed through communication from the institution, not to participate in the study. Ultimately, 240 participants registered for the study, with 187 participants completing the program by providing pre- and post-assessments. Figure 1 shows the CONSORT flow chart.

### Intervention

All components of the study were entirely virtual for convenience. The first group of students, G20, started at week 0, and the second group, G14, started from week 7, and both groups continued till week 20. From week 1 to week 12, there were online sessions, and after that, the participants were requested to follow the practice of Heartfulness Meditation on their own from week 13 to week 20. Participants registering for the study received an email from the research coordinator with instructions to study enrolment and onboarding.

### Heartfulness meditation intervention

Participants in this group were invited to participate in an online meditation program offered by the Heartfulness Institute, conducted from December 2021 through April 2022. The intervention comprised two main components:

#### Weekly live online session (60 min)

Each week, the research coordinator circulated a reminder email to all participants in the intervention group with a Zoom video conference link to join the live online meditation session, along with preparation materials specific to the corresponding module for the weekly session.

Certified Heartfulness Trainers conducted the online sessions, and the subjects were either aggregated in a classroom or from their residence at their convenience. The sessions typically included a presentation followed by a meditation session of about 15–25 min every week for 12 weeks. The session also set aside about 10–15 min for clarifications, Q&A, comments, etc., on the presentation and their experience during the meditation. The experiential aspect included practical guided meditation where participants were requested to sit comfortably and gently focus their attention, with eyes closed, on the source of light within the heart. The participants were instructed not to visualize the light but to tune in to their hearts and be open to any experience that they may have. If their attention drifted during the meditation, they were advised to redirect their attention towards the heart gently.

#### Daily practice (35 min)

Participants were requested to independently follow Heartfulness core practices (meditation, rejuvenation, and bed-time relaxation and meditation) for 35 min every day in the following schedule during the intervention period:

- 15 min Heartfulness meditation in the morning
- 15 min of Heartfulness rejuvenation in the evening
- 5 min Heartfulness bed-time relaxation and meditation before sleeping

Participants did not incur any fees or expenses associated with the meditation program and did not receive any incentives for study participation.

### Data collection

All participants completed questionnaires on demographics, Perceived Stress Scale (PSS), and Satisfaction with Life Scale (SWLS) at baseline and the end of the study period at 20 weeks. These scales were specifically selected as they have been widely used and have been validated for consistency and relevance over the years across many regions in the globe and across many cross-sections of the society.

#### Perceived stress scale

PSS (40) is a classic stress assessment instrument. Originally developed in 1983, the tool remains a popular choice to understand how different situations affect feelings and perceived stress. The questions in this scale ask about feelings and thoughts during the last month. In each case, the participants will be asked to indicate how often they felt or thought a certain way.

#### Satisfaction with life scale

SWLS (41) is a validated five-statement survey providing an overall judgment of life to measure life satisfaction. The five statements of the SWLS were answered using a 7-point Likert scale.

### Statistical analysis

Descriptive statistics were used to assess participant demographics and clinical characteristics. Changes in PSS scores and SWLS scores, pre and post intervention, were analyzed by paired-sample *t*-test, and α < 0.05 was considered statistically significant. Comparisons were made pre- and post-scores of the same groups, between the G20 and the G14 groups for both PSS and SWLS. ANOVA between age and the intercept was also done for the G20 and the G14 groups. Pearson Correlation Coefficient was used to study the relationships between the pre- and post-scores for PSS and SWLS. Statistics were calculated using SPSSv.24 (IBM, Armonk, NY).

## Results

Two hundred and forty participants registered for the study, and one hundred eighty-seven met the inclusion criteria for the analysis by completing baseline and post-intervention assessments. Participants mean age was 21.98 (SD = 4.33). The maximum age was 33 years, and the minimum age was 18 years. Key outcomes of interest were mean changes, paired *t*-test, ANOVA significance values, and Correlation Coefficients of PSS and SWLS scores.

### Perceived stress scale results

**Figure 2:**
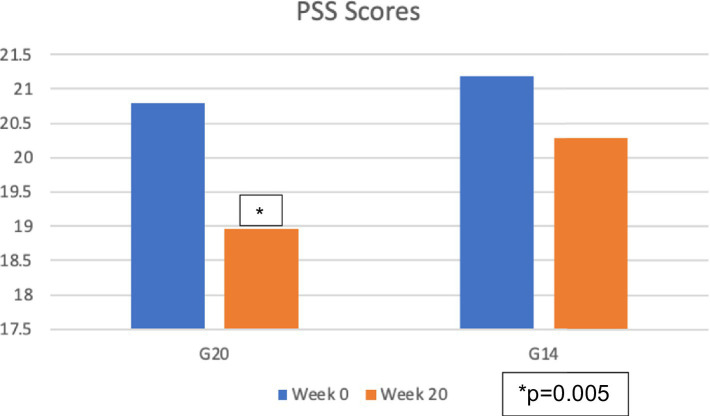
Change in the mean of the perceived stress scores (PSS).

The two groups in the Heartfulness meditation program group experienced a decrease in the mean of the PSS Scores, as indicated in Table 1. Figure 2 shows the decrease in the mean PSS score from 20.79 at week 0 to 18.96 at week 20 for G20 and from 21.18 at week 0 to 20.28 at week 20 for G14.

**Table 1:** Paired samples statistics – PSS.

		Mean	*N*	*Std. Deviation*	*Std. Error Mean*
Group 1 (G20)	Week 0 PSS	20.79	94	5.595	0.577
	Week 20 PSS	18.96	94	4.695	0.484
Group 2	Week 0 PSS	21.18	93	5.196	0.570
(G20)	Week 20 PSS	20.28	93	4.738	0.520

As indicated in Table 2, paired *t*-tests between Week 0 and Week 20 showed a significant *p*-value of 0.005 for G20 and a *p*-value of 0.157for G14.

**Table 2:** Paired Samples Test – PSS.

		Paired differences					t	df	Sig. (2-tailed)
		Mean	*Std. Deviation*	*Std. Error Mean*	95% Confidence Interval of Difference				
					Lower	Upper			
Group1(G20)	Week 0–20	1.830	6.207	0.640	0.558	3.101	2.858	93	0.005
Group 2(G14)	Week 0–20	0.904	5.761	0.632	−0.354	2.161	1.429	82	0.157

The analysis based on Pearson Correlation Coefficient for PSS is depicted in Table 3.

**Table 3:** Correlations – PSS.

		*N*	Correlation	Sig.
Group 1 (G20)	Pre-test and after 20 weeks PSS	94	0.282	0.006
Group 2 (G14)	Pre-test and after 20 weeks PSS	93	0.330	0.002

Tables 4, 5 above are the results of ANOVA analysis for age and the intercept. The *p*-value is greater than our chosen significance level (*α* = 0.05) and there seems to be no association between age and test scores for both the groups.

**Table 4:** Descriptives – PSS.

		*N*	Mean	*Std. Deviation*	*Std. Error*	95% Confidence interval for mean		Minimum	Maximum
						Lower bound	Upper bound		
Pre-test PSS Group 1 (G20)	18–25	94	21.04	5.794	0.598	19.86	22.23	6	36
	26–35	17	22.29	5.324	1.291	19.56	25.03	11	31
	Total	111	21.23	5.719	0.543	20.16	22.31	6	36
After 20 weeks PSS Group1 (G20)	18–25	89	18.58	5.047	0.535	17.52	19.65	0	32
	26–35	19	20.26	3.769	0.865	18.45	22.08	8	24
	Total	108	18.88	4.874	0.469	17.95	19.81	0	32
Pre-test PSS Group 2 (G14)	18–25	87	20.70	5.586	0.595	19.52	21.89	8	35
	26–35	23	23.70	6.138	1.280	21.04	26.35	15	38
	Total	110	21.32	5.805	0.551	20.23	22.42	8	38
After 20 weeks PSS Group 2 (G14)	18–25	64	20.36	4.735	0.592	19.18	21.54	8	32
	26–35	19	20.00	4.865	1.116	17.66	22.34	10	28
	Total	83	20.28	4.738	0.520	19.24	21.31	8	32

**Table 5:** ANOVA age and PSS.

		Sum of squares	df	Mean Square	*F*	Sig.
Week 0 PSS Group 1 (G20)	Between groups	22.551	1	22.551	0.687	0.409
	Within groups	3575.359	109	32.801		
	Total	3597.910	110			
Week 20 PSS Group 1 (G20)	Between groups	44.133	1	44.133	1.873	0.174
	Within groups	2497.302	106	23.559		
	Total	2541.435	107			
Week 0 PSS Group 2 (G14)	Between groups	163.137	1	163.137	5.019	0.027
	Within groups	3543.188	109	32.506		
	Total	3706.324	110			
Week 20 PSS Group 2 (G14)	Between groups	1.892	1	1.892	0.083	0.774
	Within groups	1838.734	81	22.700		
	Total	1840.627	82			

Since the *p*-value is less than our chosen significance level (*α* = 0.05) for both the groups, we do reject the null hypothesis. There is a weak positive correlation between pre and post scores; 0.282 for G20 and 0.330 for G14.

### Satisfaction with life results

**Figure 3:**
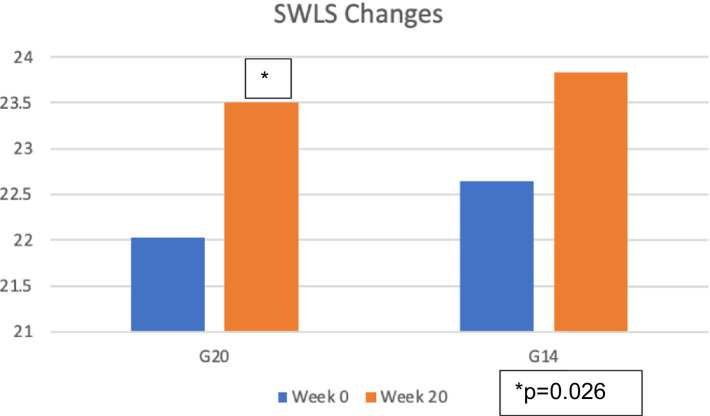
Change in the mean of satisfaction with life scores (SWLS).

The two groups in the Heartfulness meditation program group experienced an increase in the mean of the SWLS Scores as indicated in Table 6. Figure 3 shows the mean of the SWLS scores increased from 22.03 at week 0 to 23.51 at week 20 for G20 and from 22.65 at week 0 to 23.83 at week 20 for G14.

**Table 6:** Paired samples statistics – SWLS.

		Mean	*N*	*Std. Deviation*	*Std. Error Mean*
Group 1	Week 0 SWLS	22.03	94	5.941	0.613
	Week 20 SWLS	23.51	94	5.459	0.563
Group 2	Week 0 SWLS	22.65	69	6.204	0.747
	Week 20 SWLS	23.83	69	6.387	0.769

As indicated in Table 7, paired *t*-tests between Week 0 and Week 20 showed a significant *p*-value of 0.026 for G20 and a non-significant *p*-value of 0.190 for G14.

**Table 7:** Paired samples test – SWLS.

		Paired differences					*t*	df	Sig. (2-tailed)
		Mean	*Std. Deviation*	*Std. Error Mean*	95% Confidence interval of difference				
					Lower	Upper			
Group1	Pre-test – after 20 weeks SWLS	−1.479	6.336	0.654	−2.776	−0.181	−2.263	93	0.026
Group 2	Pre-test SWLS – after 20 weeks SWLS	−1.174	7.358	0.886	−2.942	0.594	−1.325	68	0.190

Tables 8, 9 above are the results of ANOVA analysis for age and the intercept. The *p*-value was greater than our chosen significance level (*α* = 0.05) and there seems to be no association between age and test scores for both the groups.

**Table 8:** Descriptives – SWLS.

		*N*	Mean	*Std. Deviation*	*Std. Error*	95% Confidence interval for mean		Minimum	Maximum
						Lower Bound	Upper Bound		
Pre-test SWLS Group 1	18–25	94	22.04	5.792	0.597	20.86	23.23	9	35
	26–35	17	22.00	6.708	1.627	18.55	25.45	8	32
	Total	111	22.04	5.908	0.561	20.92	23.15	8	35
After 20 weeks SWLS Group 1	18–25	77	23.59	5.319	0.598	22.40	24.79	11	35
	26–35	16	23.31	6.194	1.548	20.01	26.61	16	35
	Total	93	23.55	5.442	0.558	22.44	24.66	11	35
Pre-test SWLS Group 2	18–25	75	22.19	6.622	0.765	20.66	23.71	7	35
	26–35	18	22.56	6.888	1.623	19.13	25.98	8	32
	Total	93	22.26	6.638	0.688	20.89	23.63	7	35
After 20 Weeks SWLS Group 2	18–25	55	23.15	6.205	0.837	21.47	24.82	9	35
	26–35	14	26.50	6.619	1.769	22.68	30.32	13	35
	Total	69	23.83	6.387	0.769	22.29	25.36	9	35

**Table 9:** ANOVA age and SWLS.

		Sum of squares	df	Mean square	F	Sig.
Pre-test SWLS Group 1	Between groups	0.026	1	0.026	0.001	0.978
	Within groups	3839.830	109	35.228		
	Total	3839.856	110			
After 20 weeks SWLS Group 1	Between groups	1.061	1	1.061	0.035	0.851
	Within groups	2782.475	93	29.919		
	Total	2783.537	94			
Pre-test SWLS Group 2	Between groups	1.975	1	1.975	0.044	0.834
	Within groups	4051.831	91	44.526		
	Total	4053.806	92			
After 20 weeks SWLS Group 2	Between groups	125.577	1	125.577	3.177	0.079
	Within groups	2648.336	67	39.527		
	Total	2773.913	68			

The analysis based on Pearson Correlation Coefficient for SWLS is depicted in Table 10.

**Table 10:** Samples correlations - SWLS.

		*N*	Correlation	Sig.
Group 1	Pre-test and after 20 weeks SWLS	94	0.385	0.000
Group 2	Pre-test and after 20 weeks SWLS	69	0.317	0.008

Since the *p*-value is less than our chosen significance level (*α* = 0.05) for both the groups, we do reject the null hypothesis. There is a weak positive correlation between pre and post scores; 0.385 for G20 and 0.317 for G14.

## Engagement

Of the 240 participants registered in the study, 187 participants completed the intervention and completed the pre and post surveys with 53 participants lost to follow up (about 22%). As per the design of the protocol, the second group of participants were added in week 7. Table 11 depicts the average attendance at the daily morning and evening practice sessions during week 1 to week 12. After week 12 there were no formal sessions, and the participants were allowed to continue the practice at their residence.

**Table 11:** Weekly Participation of Subjects.

Month	Week No.	Avg. attendance – morning and evening sessions
December, 2021	1	54.43
January, 2022	2	44.79
	3	42.36
	4	36.71
	5	36.43
	6	29.57
February, 2022	7	57.36
	8	48.79
	9	43.29
	10	41.36
March, 2022	11	32.50
	12	27.42
Post week 12 attendance was not tracked, the practice was done at the residence of participants.		

## Discussion

Our study showed a reduction in stress levels and an improvement in satisfaction with life in female students who were willing to engage in a meditation program. The G20 group that had a longer intervention experienced a statistically significant decrease in PSS and a statistically significant increase in SWLS scores demonstrating favorable results of the Heartfulness meditation program. However, for the students in G14 the group, which underwent the intervention for a shorter duration, there was a positive impact in the PSS and SWLS mean values. But the paired *t*-tests indicated that it was not statistically significant in the pre-post scores. All other factors being same, the main difference between the two groups was that the G20 group had more exposure to the guided intervention with Heartfulness trainers compared to the G14 group. This seems to have had an impact in the scores for both PSS and SWLS. The positive but statistically insignificant changes in PSS and SWLS for the students in G14 group could have been because of the pressures and tensions during the exams and subsequent announcement of the results which overlapped with the program period. However, the impact of the stress due to exams was significantly lower for the G20 group which had a longer exposure to Heartfulness Meditation sessions with Certified Heartfulness Trainers.

Another important observation which has emerged from this study is that there is a strong correlation between perceived stress and satisfaction of life. A decreasing trend in perceived stress has shown an increasing trend in satisfaction with life. With the global increase in the mental health problems, various means to tackle it needs to be explored and meditation-based interventions could be one of them.

In comparison with other studies which had an attrition rate of 40–60%, the attrition of the subjects during this study was low at 22% (28, 31). This could be attributed to a classroom setup of offerings and the regular reminders for participation in the sessions. Previous studies involving Heartfulness meditation showed a reduction in burnout, loneliness, and improvement in sleep in mixed populations (21, 33, 42). This study is unique in the fact that it was studied in female population. In a country where gender disparities are still high, this study makes highlights the importance of addressing stress and wellbeing in female students. Meditation practices have been increasingly used for stress reduction and novel methods using virtual platforms could be a potential way to offer these interventions (19, 26, 29).

Further investigation is needed to explore reasons for this decreasing rate of participation in the practices which are very crucial in contemplative practices like meditation, especially for online-based meditation interventions. As per the prescribed practice of the intervention, daily and regular practice of the prescribed system is essential to realize the full benefit of the intervention and it seemed to be lacking (37–39). This lower level of participation as time progressed, could have been an additional factor in the G14 group not realizing the effects of the intervention. Further studies could be undertaken to examine this factor of regularity in practice and its possible impact on the effectiveness of the intervention.

Heartfulness meditation sessions conducted over via virtual platform appear to be a feasible intervention for students and could be a potential offering for improving wellbeing and reduction of stress.

## Limitations

One of the limitations of this study is that at the time of study the COVID 19 pandemic across the globe might have played a part in changes to perceived stress and satisfaction with life score. The study did not have a control group that did not receive any intervention, which would have allowed for a better comparison of the effects of the meditation program. Another limitation is that the subjects were from a single academic institution, and this could have limited randomization which in turn could have prone to confounding. The parameters could have also been affected due to exams and subsequent result declaration during the period of the study. There were no available data regarding the adherence to practice after the online sessions were completed. There could have been personal life factors of participants that played a role in the changes noticed. The long-term data about the sustained improvement to perceived stress and satisfaction with life beyond the study period has not been collected. The study relied on self-reported measures of PSS and SWLS, which might have been subject to bias or error. Additional limitation is the lack of biomarkers or physiological markers of stress or well-being that could have provided more objective and comprehensive data on the impact of the intervention.

## Conclusion

The current study is an attempt of a meditation-based intervention to improve perceived stress and satisfaction with life among female students whose literacy levels compared to male students are low in India. The results of this study indicate that a sustained practice of Heartfulness Meditation for a longer duration with Certified Heartfulness Trainer could lead to a statistically significant improvements as observed in the G20 group. While there were improvements in the mean values of PSS and SWLS noted in the G14 group, the results were not statistically significant, indicating that a sustained exposure to the practice is needed to confer larger benefits of the meditative practice. Similarly, there seems to be a strong correlation between PSS and SWLS. Decreasing score of PSS seems to increase the SWLS scores. Further larger randomized studies of similar nature can be taken up to reconfirm the findings of this study.

## Data availability statement

The raw data supporting the conclusions of this article will be made available by the authors, without undue reservation.

## Ethics statement

The studies involving humans were approved by the organizational ethics review board of the Satyam Group of Institutions and affiliated with SNDT Women’s University, Mumbai, Study ID 0508/Research/SGI-HFN. The studies were conducted in accordance with the local legislation and institutional requirements. The participants provided their written informed consent to participate in this study.

## Author contributions

All authors listed have made a substantial, direct, and intellectual contribution to the work and approved it for publication.
